# Effect of pregnancy intention, postnatal depressive symptoms and social support on early childhood stunting: findings from India

**DOI:** 10.1186/s12884-016-0909-9

**Published:** 2016-05-16

**Authors:** Ashish Kumar Upadhyay, Swati Srivastava

**Affiliations:** International Institute for Population Sciences, Govandi Station Road, Deonar, Mumbai 400088 India

**Keywords:** Pregnancy intention, Postnatal depressive symptoms, Social support, Childhood stunting, Young Lives Study, India

## Abstract

**Background:**

According to United Nation Children’s Fund, it has been estimated that worldwide about 165 million children were stunted in 2012 and India alone accounts for 38 % of global burden of stunting. This study aims to examine the effect of pregnancy intention and maternal postnatal depressive symptoms on early childhood stunting in India. We hypothesized that effect of pregnancy intention and postnatal depressive symptoms were mediated by social support.

**Methods:**

We used data from the first wave of Young Lives Study India. Multivariate logistic regression models (using generalized estimation equation) were used to examine the effect of pregnancy intention and postnatal depressive symptoms on early childhood stunting among children aged 5–21 months. The analysis included 1833 children (out of 2011 sample children) that had complete information on pregnancy intention, maternal depression and other variables.

**Results:**

Bivariate results indicate that a higher percent of children born after unintended pregnancy (40 %) were stunted than children of intended pregnancy (26 %). Likewise, the proportion of stunted children was also higher among women with high postnatal depressive symptoms (35 %) than the low level of depression (24 %). Results of multivariate logistic regression model indicate that children born after unintended pregnancy were significantly more likely to be stunted than children born after intended pregnancy (AOR: 1.76, CI: 1.25, 2.48). Similarly, early childhood stunting was also associated with maternal postnatal depressive symptoms (AOR: 1.53, CI: 1.21, 1.92). Moreover, the effect of pregnancy intention and postnatal depressive symptoms on early childhood stunting were not mediated by social support.

**Conclusions:**

The findings of this study provide conclusive evidence regarding consequences of pregnancy intention and postnatal depressive symptoms on early childhood stunting in India. Therefore, there is a need to identify the women with unintended pregnancy and incorporate the promotion of mental health into their national reproductive and child health programme.

## Background

Childhood stunting, defined as height for age z-score below -2sd of the median of reference population by the World Health Organization, is remains one of the important public health concern throughout the developing countries [1]. According to United Nation Children’s Fund (UNICEF), it has been estimated that worldwide about 165 million children are stunted in 2012 and India alone accounts for 38 % of global burden of stunting in the world [2]. In terms of incidence, India accounts for more than 60 million stunted children worldwide [2]. Recent data from third Indian National Family Health Survey (INFHS) suggest that about 45 % children under age 3 year are stunted [3]. Such a high prevalence of stunting is a matter of serious concern because stunted children are associated with weaker immune system, higher risk of developing diarrheal disease, acute respiratory infection, delay in motor skills, cognitive, and social development during childhood [4–12] and more likely to suffer from high blood pressure, obesity, diabetes and heart disease during adulthood [6, 13–15]. Another concern related to stunting is it passes from one generation to another as a grim inheritance [16, 17].

Studies have identified several risk factors such as birth size, mother’s education, mother’s age at birth of the child, consumption of iron folic acid tablet during pregnancy and socio economic condition for childhood stunting in developing countries [18–20]. Studies from India also reported that birth size, mother’s education, antenatal check-up during pregnancy, serious illness, drinking water, toilet facility, economic status, place of residence are statistically associated with childhood stunting [21–23]. A study by World Health Organization (WHO) suggests that about 50 % of childhood stunting in India is related with repeated diarrhoea or intestinal worm infection from unsafe water and poor sanitation or hygiene [24]. However, very few studies have analysed the cumulative effect of both pregnancy intention and postnatal depressive symptoms on early childhood stunting simultaneously in developing countries and perhaps there was no such study in India.

Pregnancy intention may influence child health outcomes through increased level of depression, change in behaviour in parenting, had a greater risk of smoking, consumption of alcohol, and were less likely to attend antenatal care and to take iron folic tablets during pregnancy [25–28]. Which in turn increase the risk of preterm birth, low birth weight, and of not receiving sufficient resources for healthy development [29, 30]. Existing literature on the association between pregnancy intention and childhood stunting has been inconsistent. A number of studies from developing countries reported that pregnancy intention is linked to childhood stunting [22, 31–33]. On the other hand, a few studies reported that pregnancy intention is not associated with childhood stunting in developing countries [31, 33]. A study using cross-sectional data from INFHS, reported that unintended birth was disadvantage in terms of childhood stunting [22]. However, the majority of these studies did not adjust for psychosocial factors such as mental depression and social support.

Another subject worth exploring childhood stunting is postnatal depressive symptoms. In the context of developing countries poor maternal mental health, in particular, maternal depression may be a significant risk factor for poor growth during early childhood. In addition to depression, women are particularly prone in postpartum period because of hormonal changes associated with childbirth and stressor related to parenting [34–36]. The combination of women vulnerability to mental depression and responsibility for child care in developing countries means that maternal mental health could have a significant effect on growth during early childhood [37]. A number of previous studies from developing countries have shown that maternal depressive symptoms were significantly associated with childhood stunting [35, 38–43]. A study from Goa (India) also shows that children of the depressed mother were 2.3 times more likely to be stunted than children of non-depressed mothers [41]. A similar finding was also reported by another study from the rural area of Tamilnadu (India) [44]. However, these studies in India were based on small sample sizes and lacked representativeness.

The association between pregnancy intention, maternal depression, social support and child health outcome is undoubtedly complex [45–48]. Unintended pregnancy may increase a women’s exposure to the psychosocial stressor, decrease social support provided to her by the partner, increase her level of depressive symptoms and decrease her overall life satisfaction [46]. However, social support has potential to play protective effect by buffering the impact of life stress on the well-being of the women during pregnancy [49–52]. Studies have shown that social supports play a buffering role from stressful life events by providing resources, support and strength during pregnancy [46, 53–57]. In an analysis of YLS data from four developing countries, De Silva and Harpham [58] found that support from the individual was linked to child nutritional status in Ethiopia, Peru and Vietnam. The only one study from Ethiopia reported that effect of maternal depression on child birth outcome was mediated by social support [57]. However, no such study exist from India. Therefore, in this study, we examined whether women who reported unintended pregnancy or who experienced postnatal depression are at higher risk of having stunted children in India. We hypothesized that effect of pregnancy intention and maternal postnatal depressive symptoms on childhood stunting was mediated by social support.

## Methods

### Study setting and sample

We used the data from the first waves of the Young Lives Study (YLS), which was conducted in the state of Andhra Pradesh in India during 2002. Young Lives is an international longitudinal study investigating the changing nature of childhood poverty. About 12000 children are being followed in four countries: Ethiopia, Peru, Vietnam and India (Andhra Pradesh). Each country has two cohorts: younger cohort and older cohort. The younger cohort consists of about 2000 children born during 2001–2002 and the older cohort consists of about 1000 children born during 1994–1995 to be followed over a period of 15 years [59, 60]. The YLS is conducted every 3/4 years to collect data on a range of indicators related to the growth and development of children. YLS collects information on child welfare outcomes including nutritional status, growth, physical health, cognitive development, social and emotional well-being and educational development [59–61].

A multistage sampling design was adopted in YLS. In the first stage, two districts were selected from each of the three geographic regions (Coastal, Rayalaseema and Telangana) of Andhra Pradesh. In the second stage, 19 (15 from rural areas and 4 from urban areas) sentinel sites (administrative blocks or ‘mandals’) were selected from the six selected districts. In addition, one sentinel site was selected from the urban slums of the Hyderabad city. In the third stage, villages were selected from rural sentinel sites and wards were selected from the urban sites. All the households with 5–21 months old child (born during 2001–2002) or 87–103 months old child (born during 1994–95) in the selected villages and wards were included in YLS. Overall, 2011 households (with 2011 children) in the younger cohort and 1008 households (with 1008 children) in the older cohort were included in the first wave of YLS. (for details of YLS sampling design, see [59, 60, 62]). As the objective of this study is to examine the effects of pregnancy intention, postnatal depressive symptoms and social support on early childhood stunting, we used data from the first wave of the younger cohort (aged 5–21 months) born during 2000–2001.

The study included only those children where the mother was the primary caregiver of that child. Individuals with missing data on any of the variable included in the model were excluded based on the following criteria: if the respondent was not biological mother or mother died, women unsure about their pregnancy intention, information on postnatal depression could not be collected and child height could not be measured. This resulted in a 9 % of the sample being excluded from the analysis. Of the 2011 children, 1833 (91 %) children whose complete information on pregnancy intention, postnatal depression, social support, height for age z-score and other variables was collected were considered for the study.

### Outcome variable

The outcome variable of interest is early childhood stunting. Children with height-for-age Z-score (HAZ) below minus two standard deviations (HAZ < -2SD) from the median of the reference population were considered short for their age or stunted. Such children are also considered chronically malnourished [63]. Children with height for age Z-score below -6 or above 6 were excluded from the analysis (biologically implausible value defined by WHO) [1].

### Key independent variables

The key variables of interest are: pregnancy intention (intended, unintended), postnatal depressive symptoms (non-cases, cases) and social support (low, medium, high).

The survey collected data on mother’s pregnancy intention of the index child. The survey asked women “At the time you became pregnant with index child, did you want to become pregnant”. If women reported “yes’ then it was coded as intended pregnancy and if the response was “no” i.e, mistimed/unwanted, then it was coded as unintended pregnancy.

The information on maternal postnatal depressive symptoms was collected soon after the birth of index child. The postnatal depressive symptoms were measured using WHO recommended tools of self-reported 20 items (SRQ20) that consists 20 questions and answer of each question were reported in yes/no/don’t know with a reference period of last 30 days [64]. We count the number of ‘yes’ responses to the 20 questions. If there are 8 or more ‘yes’ responses, then it was classified as a case and less than 8 ‘yes’ responses were classified as non case. The cut of score to determine how many ‘yes’ responses constitute a case have been validated against clinical assessment [65, 66].

The information on any kind of economic support, emotional support or assistance was also collected in the survey. The survey asked women, since last 12 months, did they receive any kind of economic help, emotional help or assistance from work related/trade union (yes, no), community association/co-op (yes, no), women’s group (yes, no), political group(yes, no), religious group (yes, no), credit or funeral group (yes, no), sports group (yes, no), family (yes, no), neighbourhood (yes, no), friends (yes, no), community leaders (yes, no), religious leader (yes, no), politicians (yes, no), government official/civil service (yes, no), charitable organization/NGO (yes, no) and other (yes, no). If women received any kind of help from afore-mentioned group or person, then it was coded as ‘1’ and ‘0’ otherwise. Further, help from each group/person added together which ranges from 0 to 16. If a women reported no help or assistance, then it was coded as ‘low’ social support. If the number of supports ranges from 1 to 4, it was considered as ‘medium’ social support and from 5 to 16 were considered as high social support. The details of the description of social support measurement are presented elsewhere [67].

### Other variables

A number of other socio-economic, demographic and residence related variables have also been shown to have the significant effect on childhood stunting. Accordingly we included birth size (average and above, below average), preterm birth (full term, 1–2 week earlier, 3 or more week earlier), age of the child (in months), sex of the child (male, female), ever breastfed (no, yes), serious illness (no, yes), mother’s age at birth of child (<18 years, 18–24 years, 25–29 years, > = 30 years), mother’s education (below primary, primary and above), mother’s working status (not working, agricultural work, others), ante-natal check-ups (no ANC, <4 ANC, > = 4ANC), iron folic acid tablets (no IFA, <90 IFA, > = 90 IFA),tetanus injection (<2 TT, > = 2 TT), household head’s education (below primary, primary and above), sex of the household head (male, female), household size, wealth index (poor, middle, rich), drinking water (improved, non-improved), toilet facility (improved, non-improved), income shocks (no, yes), religion (hindu, muslim, others), caste (scheduled tribes, scheduled caste, other backward caste, others) and place of residence (rural, urban).

The survey collected data on respondent’s (mother/caregiver) perception about size of the baby at birth. YLS asked the respondent when child was born he/she was very small, small, average, large or very large? Very small or small size at birth was coded as ‘below average’, and average, large and very large size at birth was coded as ‘average and above average’.

The information on serious illnesses were also collected during the survey. The survey asked mother/caregiver since the birth of child, whether he/she had any serious illnesses or injury when she thought child might be died? (Yes/No/Don’t know).

The wealth index was calculated using wealth score, which are already computed and given in the YLS dataset. The wealth score was generated through a principle component analysis conducted on a set of variables based on household assets (including radio, refrigerator, bicycle, television, motorbike/scooter, car, pump, sewing machine, mobile, phone, landline telephone, fan, almirah, clock, table, chair, sofa, bedsheet and animals), household quality (including wall, roof and floor) and services (including electricity, drinking water, toilet facility). The lowest 33.3 % households were coded as poor, the next 33.3 % as middle and the remaining 33.3 % as rich.

YLS also collected information on main source of drinking water. Children were classified into two categories according to whether household relied on safe or unsafe water supply for their drinking. Households having piped water into dwelling/yard/plot or using public tap/standpipe or using tube well/borehole or protected dug well were considered as using safe drinking water. Other households were categorized as using unsafe drinking water. Information on type of toilet facility used by household was also gathered in YLS. Improved toilet facilities include flush toilet/pit latrine connected to septic tank. Non-improved toilet facilities include public/shared facility, simple latrine, and toilet in health post or forest/field/open place.

Income shock refers to any events or big changes that significantly reduce the economic welfare of household. Income shocks at the household level were accessed by the answer to the following question in the YLS:

Since birth of child, whether the household suffered from natural disaster (yes; no), decrease in food availability (yes; no), livestock died (yes; no), crop failed (yes; no), job loss (yes; no), serious illness/injury (yes; no) and victim of crime (yes; no). If household suffered from any one of afore-mentioned event were coded as ‘1’ otherwise, ‘0’.

### Statistical analysis

Bivariate analysis was done to compare early childhood stunting by sample characteristics using cross tabulation. Further, we used logistic regression model (using generalized estimation equations to take into account the cluster nature of sample) to examine the effect of pregnancy intention, postnatal depressive symptoms and social support on stunting among children aged 5–21 months. Firstly, the unadjusted association between key independent variables and outcome variable was estimated, followed by the association adjusted for potential confounding variables. Unadjusted and adjusted odds ratio and 95 % confidence interval were reported. Moreover, to access whether social support mediates the effects of pregnancy intention and postnatal depressive symptoms on childhood stunting, we used recommended procedure [68]. Variables were included into the multivariate model based on previous studies and their association with childhood stunting in bivariate analysis. All the variables were tested for multi-collinearity using variance inflammation factor (VIF) before being included in the regression models. All the statistical computations were done in STATA 13.0.

## Results

The characteristics of the study population are shown in Table 1. The result shows that about 8 % of women reported that their pregnancy was unintended or mistimed while the prevalence of postnatal depressive symptoms was about 30 %. The prevalence of stunting was about 27 % among children aged 5 to 21 months. A higher proportion of children were stunted of whose mothers had unintended pregnancy (40 %) than those who had intended pregnancy (26 %). Similarly, childhood stunting was also higher among those whose mothers had symptoms of postnatal depression (35 %) than those who had no symptoms of postnatal depression (24 %). The percentage of stunted children did not vary substantially according to the social support.

**Table 1:** Percent distribution of childhood stunting by pregnancy intention, postnatal depressive symptoms, social support and social-demographic factors among children aged 5–21 month in India

Background characteristics		Sample Size (N)	Proportion	Not Stunted	Stunted	Chi-2 (*p*-value)
Pregnancy Intention	Intended	1684	91.9	73.9	26.1	12.67(0.00)
	Unintended	149	8.1	60.4	39.6	
Maternal Depression	Non cases	1286	70.2	76.3	23.7	25.94(0.00)
	Cases	547	29.8	64.7	35.3	
Social Support	Low	356	19.4	75.6	24.4	1.70(0.426)
	Medium	1428	77.9	72.1	27.9	
	High	49	2.7	73.5	26.5	
Birth Size	Average and above	1373	74.9	76.5	23.5	36.7(0.00)
	Below Average	460	25.1	62.0	38.0	
Pre-mature birth	Full-term	1678	91.5	73.6	26.4	8.8(0.01)
	1–2 weak earlier	108	5.9	68.5	31.5	
	3 or more week earlier	47	2.6	55.3	44.7	
Sex of child	Male	985	53.7	71.9	28.1	0.98(0.32)
	Female	848	46.3	73.9	26.1	
Ever breastfeed	No	49	2.7	77.6	22.5	0.57(0.45)
	Yes	1784	97.3	72.7	27.3	
Serious Illness	No	1417	77.3	74.7	25.3	11.4(0.00)
	Yes	416	22.7	66.4	33.7	
Mother’s education	Below primary	1091	59.5	67.7	32.3	35.4(0.00)
	Primary and above	742	40.5	80.3	19.7	
Mother’s working status	Not working	920	50.2	77.4	22.6	20.6(0.00)
	Agricultural work	731	39.9	67.4	32.6	
	Other work	182	9.9	71.4	28.6	
Mother’s age at birth of child	<18 year	146	8.0	61.6	38.4	11.0(0.01)
	18–24 year	1270	69.3	73.5	26.5	
	25–29 year	320	17.5	75.6	24.4	
	> = 30 year	97	5.3	71.1	28.9	
Ante natal check-up	No ANC	199	10.9	70.4	29.7	3.4(0.18)
	<4 ANC	483	26.4	68.1	31.9	
	> = 4ANC	1151	62.8	75.2	24.8	
Iron folic acid tablet	No IFA	287	15.7	69.0	31.0	9.4(0.01)
	<90 IFA	116	6.3	69.8	30.2	
	> = 90 IFA	1430	78.0	72.8	26.2	
Tetanus Injection	<2 TT	235	12.8	69.4	30.6	1.6(0.20)
	> = 2TT	1598	87.2	73.3	26.7	
Household’s head education	Below primary	1068	58.3	68.4	31.7	25.9(0.00)
	Primary and above	765	41.7	79.1	20.9	
Sex of head of household	Male	1675	91.4	73.2	26.8	1.3(0.26)
	Female	158	8.6	69.0	31.0	
	Household size					
Wealth index	Poor	606	33.1	63.2	36.8	55.0(0.00)
	Middle	604	33.0	73.0	27.0	
	Rich	623	34.0	82.0	18.0	
Drinking water	Improved	1536	83.8	73.1	26.9	0.37(0.54)
	Non improved	297	16.2	71.4	28.6	
Toilet facility	Improved	484	26.4	82.9	17.2	33.4(0.00)
	Non-improved	1349	73.6	69.2	30.8	
Income shocks	No shocks	1064	58.0	73.7	26.3	0.93(0.33)
	At least one shocks	769	42.0	71.7	28.4	
Religion	Hindu	1606	87.6	72.4	27.7	4.4(0.11)
	Muslim	133	7.3	80.5	19.6	
	Others	94	5.1	70.2	29.8	
Caste	Schedule tribes	264	14.4	62.1	37.9	46.5(0.00)
	Schedule caste	325	17.7	65.5	34.5	
	Other backward caste	854	46.6	74.1	25.9	
	Others	390	21.3	83.3	16.7	
Place of residence	Rural	1364	74.4	69.6	30.4	28.6(0.00)
	Urban	469	25.6	82.3	17.7	
	Total	1833	100.0	72.8	27.2	

Prevalence of stunting was higher among children of small birth size than average/above average birth size (38 % against 23 %), those who born preterm compared with term babies (45 % against 26 %) and those who suffered from serious illness/injury during early childhood compared to those who did not suffer from any illness/injury (34 % against 25 %). A higher proportion of children were stunted of those women who had less schooling and who had the lower age at birth of the child. Likewise, the prevalence of stunting was higher in children, among mothers who did not go for the antenatal check-up, consume less than 90 IFA tablet and had less than 2 TT injections during their pregnancy. The prevalence of stunting also varied substantially according to the socioeconomic status of the household and rural-urban residence. Prevalence of stunting was higher in children from household using non-improved toilet facility, belongs to poor wealth quintiles and those who are living in rural area. Results of the logistic regression model to examine the effects of pregnancy intention, postnatal depressive symptoms and social support on childhood stunting are shown in Table 2. Results of unadjusted logistic regression model shows that among socio-demographic and psychosocial factors; pregnancy intention, postnatal depressive symptoms, birth size, preterm birth, serious illness, mother’s education, mother’s age at birth of child, education of household’s head, wealth index, toilet facility and place of residence were statistically associated with childhood stunting. The result of adjusted logistic regression model also shows that pregnancy intention and postnatal depressive symptoms were statistically associated with childhood stunting, after controlling social supports and well-known determinants of stunting. Children born after unintended pregnancy were 1.76 times (AOR- 1.76; 95 % CI: 1.25–2.48) more likely to be stunted than children born after intended pregnancy. Likewise, children of those women who showed symptoms of postnatal depression were 1.53times (AOR-1.53; 95 % CI: 1.21–1.92) more likely to be stunted than non-depressed women. Although, in the adjusted model, pregnancy intention and postnatal depressive symptoms was statistically associated with childhood stunting while the magnitude of the effects of pregnancy intention and postnatal depressive symptoms on childhood stunting was attenuated. In order to examine whether the effect of pregnancy intention and postnatal maternal depressive symptoms on early childhood stunting were mediated by the presence of social supports, we used a series of multivariate logistic regression models. Multivariate models I and II shows that unintended pregnancy was significantly associated with childhood stunting even after controlling social support and other socioeconomic and residence related variables (Table 3). Likewise, multivariate models III and IV shows that postnatal depressive symptoms are statistically associated with childhood stunting after adjustment of social support and other socioeconomic and residence related variables (Table 4). Notably, the magnitude of the effect of pregnancy intention and postnatal maternal depressive symptoms on childhood stunting was not changed even after including the social support. Social support was not statistically associated with childhood stunting. Apart from these variables some other variables are also significantly associated with childhood stunting in the multivariate model. Children of small birth size than average or above birth size, preterm birth compare to the term baby, lower age of mother at birth of the child and belong to lower wealth quintiles were more likely to be stunted than their counterpart children (Table 2).

**Table 2:** Results of logistic regression analysis showing effect of pregnancy intention, postnatal depressive symptoms and social support on stunting among children aged 5–21 months in India

	Characteristics	Unadjusted Odds Ratio (95%C.I.)	Adjusted Odds Ratio (95%C.I.)
Pregnancy Intention	Intended®		
	Unintended	1.86* (1.35,2.55)	1.76*(1.25,2.48)
Maternal Depression	Non cases®		
	Cases	1.75* (1.42,2.16)	1.53*(1.21,1.92)
Social Support	Low®		
	Medium	1.19 (0.91,1.57)	1.26(0.94,1.68)
	High	1.12 (0.56,2.22)	1.58(0.80,3.11)
Birth Size	Average and above®		
	Below Average	2.00* (1.61,2.47)	1.82*(1.44,2.30)
Pre-mature birth	Full-term®		
	1–2 weak earlier	1.28 (0.85,1.92)	1.61*(1.04,2.49)
	3 or more week earlier	2.25* (1.33,3.81)	2.23*(1.22,4.05)
	Age (in months)	1.12* (1.09,1.16)	1.14*(1.10,1.18)
Sex of child	Male®		
	Female	0.90 (0.73,1.11)	0.88(0.71,1.09)
Ever breastfeeding	No®		
	Yes	1.30 (0.63, 2.67)	1.08(0.53,2.18)
Serious Illness	No®		
	Yes	1.50* (1.20,1.88)	1.24(0.97,1.58)
Mother’s education	Below primary®		
	Primary and above	0.51* (0.41, 0.65)	0.80(0.61,1.06)
Mother’s working status	Not working®		
	Agricultural work	1.65* (1.33,2.06)	1.00 (0.77,1.30)
	Other work	1.37 (0.96,1.95)	1.03(0.72,1.48)
Mother’s age at birth of child	<18 year®		
	18–24 year	0.58* (0.41,0.80)	0.60*(0.43,0.86)
	25–29 year	0.52* (0.35,0.77)	0.56*(0.37,0.85)
	> = 30 year	0.65 (0.38,1.10)	0.60(0.34,1.05)
Ante natal check-up	No ANC®		
	<4 ANC	1.11 (0.78,1.57)	1.45(0.68,3.07)
	> = 4ANC	0.78 (0.56,1.08)	1.20(0.56,2.57)
Iron folic acid tablet	No IFA®		
	<90 IFA	0.96 (0.61,1.51)	0.96(0.53,1.76)
	> = 90 IFA	0.79 (0.60,1.03)	1.04(0.65,1.67)
Tetanus Injection	<2 TT®		
	> = 2TT	0.82 (0.61,1.10)	0.86(0.41,1.80)
Household’s head education	Below primary®		
	Primary and above	0.57* (0.46,0.71)	0.76*(0.58,0.99)
Sex of head of household	Male®		
	Female	1.23 (0.87,1.72)	1.21(0.82,1.78)
	Household size	1.00 (0.96,1.05)	1.03(0.99,1.08)
Wealth index	Poor®		
	Middle	0.63* (0.50,0.80)	0.72*(0.55,0.93)
	Rich	0.38* (0.28,0.50)	0.60*(0.39,0.91)
Drinking water	Improved®		
	Non improved	1.09 (0.83, 1.43)	0.85(0.63,1.15)
Toilet facility	Improved®		
	Non-improved	2.14* (1.59,2.89)	0.85(0.52,1.40)
Income socks	No socks®		
	At least one socks	1.11 (0.90,1.36)	0.77*(0.60,0.99)
Religion	Hindu®		
	Muslim	0.63 (0.39,1.04)	1.12(0.65,1.95)
	Others	1.11 (0.71,1.73)	0.92(0.56,1.50)
Caste	Schedule tribes®		
	Schedule caste	0.86 (0.63,1.17)	0.97(0.69,1.37)
	Other backward caste	0.57* (0.43,0.75)	0.70*(0.51,0.95)
	others	0.33* (0.22,0.48)	0.44*(0.28,0.71)
Place of residence	Rural®		
	Urban	0.49* (0.36,0.66)	1.02(0.63,1.64)

® reference, * significant at 5 %

**Table 3:** Results of multivariate logistic regression models to access whether social supports mediate the effect of pregnancy intention on stunting among children aged 5–21 months in India

	Background Characteristics	Adjusted Odds Ratio (95 % C.I.)^ǂ^	Adjusted Odds Ratio (95 % C.I.)
		Model-I	Model-II
Pregnancy Intention	Intended®		
	Unintended	1.75*(1.25,2.47)	1.76*(1.25,2.47)
Social Support	Low®		
	Medium		1.24(0.93,1.65)
	High		1.45(0.74,2.82)
Birth Size	Average and above®		
	Below Average	1.80*(1.43,2.27)	1.82*(1.44,2.29)
Pre-mature birth	Full-term®		
	1–2 weak earlier	1.58*(1.02,2.45)	1.63*(1.05,2.53)
	3 or more week earlier	2.22*(1.20,4.11)	2.28*(1.24,4.21)
Age (in months)	Age (in months)	1.14*(1.10,1.18)	1.14*(1.10,1.18)
Sex of child	Male®		
	Female	0.88(0.71,1.09)	0.87(0.70,1.08)
Ever breastfeeding	No®		
	Yes	0.96(0.50,1.85)	0.99(0.50,1.94)
Serious Illness	No®		
	Yes	1.28*(1.00,1.63)	1.28*(1.00,1.63)
Mother’s education	Below primary®		
	Primary and above	0.80(0.61,1.05)	0.80(0.60,1.05)
Mother’s working status	Not working®		
	Agricultural work	1.01(0.78,1.32)	1.00(0.77,1.31)
	Other work	1.05(0.74,1.51)	1.06(0.74,1.51)
Mother’s age at birth of child	<18 year®		
	18–24 year	0.64*(0.45,0.91)	0.63*(0.45,0.90)
	25–29 year	0.60*(0.40,0.91)	0.60*(0.39,0.90)
	> = 30 year	0.65(0.37,1.13)	0.65(0.37,1.13)
Ante natal check-up	No ANC®		
	<4 ANC	1.29(0.60,2.77)	1.30(0.61,2.79)
	> = 4 ANC	1.06(0.49,2.29)	1.07(0.49,2.32)
Iron folic acid tablet	No IFA®		
	<90 IFA	1.01(0.55,1.85)	1.00(0.54,1.84)
	> = 90 IFA	1.07(0.66,1.74)	1.05(0.64,1.70)
Tetanus Injection	<2 TT®		
	> = 2TT	0.88(0.41,1.85)	0.88(0.42,1.85)
Household’s head education	Below primary®		
	Primary and above	0.76*(0.58,0.99)	0.76*(0.58,0.99)
Sex of head of household	Male®		
	Female	1.18(0.81,1.72)	1.17(0.80,1.71)
Household size	Household size	1.03(0.98,1.08)	1.03(0.98,1.08)
Wealth index	Poor®		
	Middle	0.72*(0.56,0.94)	0.72*(0.55,0.93)
	Rich	0.56*(0.36,0.86)	0.55*(0.36,0.85)
Drinking water	Improved®		
	Non improved	0.83(0.61,1.12)	0.84(0.62,1.13)
Toilet facility	Improved®		
	Non-improved	0.85(0.52,1.41)	0.85(0.52,1.40)
Income socks	No socks®		
	At least one socks	0.81(0.63,1.03)	0.80(0.63,1.03)
Religion	Hindu®		
	Muslim	1.09(0.64,1.88)	1.14(0.66,1.97)
	Others	0.85(0.51,1.40)	0.88(0.53,1.45)
Caste	Schedule tribes®		
	Schedule caste	1.06(0.75,1.50)	1.04(0.74,1.47)
	Other backward caste	0.72*(0.53,0.99)	0.71*(0.52,0.98)
	Others	0.49*(0.31,0.77)	0.48*(0.30,0.76)
Place of residence	Rural®		
	Urban	0.98(0.61,1.59)	1.01(0.63,1.62)

® reference, * significant at 5 %, ǂ model not adjusted for social support

**Table 4:** Results of multivariate logistic regression models to access whether social supports mediate the effect of postnatal depressive symptoms on stunting among children aged 5–21 months in India

	Background Characteristics	Adjusted Odds Ratio (95 % C.I.)^ǂ^	Adjusted Odds Ratio (95 % C.I.)
		Model-III	Model-IV
Maternal Depression	Non cases®		
	Cases	1.51*(1.20,1.89)	1.52*(1.21,1.92)
Social Support	Low®		
	Medium		1.23(0.93,1.64)
	High		1.59(0.81,3.11)
Birth Size	Average and above®		
	Below Average	1.80*(1.43,2.27)	1.82*(1.45,2.30)
Pre-mature birth	Full-term®		
	1–2 weak earlier	1.53(0.99,2.36)	1.58*(1.02,2.45)
	3 or more week earlier	2.21*(1.20,4.05)	2.22*(1.21,4.07)
Age (in months)		1.14*(1.10,1.18)	1.14*(1.10,1.18)
Sex of child	Male®		
	Female	0.90(0.72,1.11)	0.89(0.72,1.10)
Ever breastfeeding	No®		
	Yes	1.03(0.53,2.02)	1.07(0.54,2.14)
Serious Illness	No®		
	Yes	1.25(0.98,1.59)	1.25(0.98,1.60)
Mother’s education	Below primary®		
	Primary and above	0.81(0.62,1.07)	0.81(0.61,1.06)
Mother’s working status	Not working®		
	Agricultural work	1.01(0.78,1.32)	1.01(0.77,1.31)
	Other work	1.03(0.71,1.48)	1.03(0.72,1.49)
Mother’s age at birth of child	<18 year®		
	18–24 year	0.61*(0.43,0.87)	0.61*(0.43,0.86)
	25–29 year	0.58*(0.38,0.87)	0.57*(0.38,0.87)
	> = 30 year	0.61(0.35,1.08)	0.62(0.35,1.08)
Ante natal check-up	No ANC®		
	<4 ANC	1.61(0.77,3.36)	1.62(0.78,3.37)
	> = 4ANC	1.31(0.62,2.76)	1.32(0.63,2.76)
Iron folic acid tablet	No IFA®		
	<90 IFA	0.96(0.52,1.75)	0.95(0.52,1.74)
	> = 90 IFA	1.03(0.64,1.64)	1.01(0.63,1.62)
Tetanus Injection	<2 TT®		
	> = 2TT	0.78(0.38,1.59)	0.78(0.38,1.58)
Household’s head education	Below primary®		
	Primary and above	0.77(0.59,1.00)	0.77(0.59,1.00)
Sex of head of household	Male®		
	Female	1.23(0.84,1.79)	1.22(0.84,1.78)
Household size	Household size	1.04(0.99,1.09)	1.04(0.99,1.08)
Wealth index	Poor®		
	Middle	0.74*(0.57,0.95)	0.73*(0.56,0.95)
	Rich	0.61*(0.40,0.94)	0.61*(0.40,0.93)
Drinking water	Improved®		
	Non improved	0.85(0.63,1.15)	0.87(0.64,1.17)
Toilet facility	Improved®		
	Non-improved	0.88(0.54,1.45)	0.88(0.54,1.44)
Income socks	No socks®		
	At least one socks	0.79(0.62,1.01)	0.79(0.61,1.01)
Religion	Hindu®		
	Muslim	1.06(0.62,1.82)	1.10(0.63,1.89)
	Others	0.90(0.55,1.46)	0.93(0.57,1.51)
Caste	Schedule tribes®		
	Schedule caste	0.98(0.70,1.37)	0.95(0.68,1.34)
	Other backward caste	0.71*(0.52,0.97)	0.70*(0.51,0.95)
	Others	0.47*(0.29,0.74)	0.46*(0.29,0.72)
Place of residence	Rural®		
	Urban	0.99(0.61,1.59)	1.02(0.63,1.63)

® reference, * significant at 5 %,ǂ model not adjusted for social support

## Discussion

Our findings clearly suggest that mother’s pregnancy intention and postnatal depressive symptoms are significantly associated with early childhood stunting, after adjustment of the well-known determinants of child nutritional status in India. Finding indicates that unintended births were significantly more likely to be stunted than intended during early childhood. Several previous studies reported the inconsistent association between pregnancy intention and childhood stunting [31, 69]. However, findings of this study are consistent with previous studies from India [22], Bolivia [34], Peru [31] and Bangladesh [70]. Further, the study provides a significant association between maternal postnatal depressive symptoms and childhood stunting. Our findings are consistent with previous studies conducted in developing countries [40, 41, 43]. In contrast, to these studies a few studies have reported no association between depressive symptoms and childhood stunting in developing countries [71–73]. However, the majority of these studies in developing countries did not adjust for psychosocial factors including pregnancy intention and social support. Moreover, findings indicate that effects of pregnancy intention and postnatal depression on early childhood stunting were not mediated by the social support. One of the possible reason for the lack of association between social support and childhood stunting may be reverse causality whereby women who are depressed are more likely to receive support from other individuals within their community, relatives and friends. Notably, Kawachi and Berkman [74] reported that protective effect of social support may not be uniform across society. They argue that social connection may increase the level of depression among women with low resources. A similar finding was also reported by Mitchella and LaGlory [75] in an impoverished community in the USA. Such a scenario is plausible among the poor biased YL sample. The study found significant interactions between pregnancy intention and maternal postnatal depression in predicting early childhood stunting. To the best of our knowledge, this is the first study of its kind that examined effect of pregnancy intention and maternal postnatal depression on childhood stunting in India.

This study has some limitations. First, only 8 % of the births were reported as unintended by the mothers in YLS. This figure is much lower than that obtained for Andhra Pradesh (India) from the INFHS 2005–06 (18.4 %). The difference in the estimates between the INFHS 2005–06 and YLS could be due to two reasons. First, the wordings of the questions in the two surveys are completely different. Notably, the questions on pregnancy intention in INFHS are far more comprehensive than the questions in YLS. Secondly, INFHS provides estimates for Andhra Pradesh whereas the YLS is restricted to few sentinel sites in Andhra Pradesh. Given the difference in the two estimates, the estimates of unwanted births in YLS can be safely taken as a lower bound of the actual estimate. Hence, the effects of pregnancy intention that we have obtained in our study would have been more compelling and YLS accurately captured the unintended births. Second, our study could not distinguish the separate effect of unwanted birth and mistimed birth on early childhood stunting due to data limitation. Third, our estimate of the effect of pregnancy intention on childhood stunting is likely to be lower than expected due to mortality selection. There is a higher chance of death during infancy among unwanted children as compared to wanted children [22, 23]. Our estimates of the effect of pregnancy intention on childhood stunting are also likely to be affected by differential reporting of pregnancy intention by the mother. Differential could be happened as data based on the retrospective report of intention and this method is subject to bias in recall and reporting. For example, during the survey mother may report that index child is intended at the time of conception, although at the time of conception child was unintended. Most researchers agreed that mother’s feeling and attitude regarding pregnancy intention change from unintended to intended following the births of child and hence reduces the effect of pregnancy intention on childhood stunting [32]. However, pregnancy intention was measured soon after birth (from 5 to 21 months), therefore, bias in recall and reporting should be minimal. Another concern related to quality and timing of the social support, we are only able to control for type of social support. However, quality and timing of the social support is also essential [76, 77].

Despite these limitations, our study has some strength. First, a large cohort of children included in the analysis and represents children of a wide range of family background. Second YLS is the only large scale available data that provides information on pregnancy intention, maternal postnatal depressive symptoms, social support and childhood stunting in India. Third, YLS uses a child focus mixed methods sampling approach, allow the examination of the complex interrelationship between pregnancy intention, postnatal depressive symptoms, social support and childhood stunting in India. Fourth, study includes both rural and urban areas, representing a range of regions, policy context and living condition that reflects the ethnic, geographical and religious diversity of the country. Finally, our study came out with some findings that may either lead to the formulation of new policies or may lead to the strengthening of the existing policies and programmes.

## Conclusions

Our study provides strong evidence regarding the effect of pregnancy intention and postnatal depressive symptoms on early childhood stunting in India. The findings of our study have important policy implications. The prevalence of early childhood stunting is still serious health issue in India. Recent estimates from INFHS suggest that about 48 % of children under age five are stunted in India, which results in more than 60 million of stunted children. The risk of early childhood stunting is higher among women of unintended pregnancy and those with postnatal maternal depressive symptoms in India. Therefore, there is urgent need to identify the women with unintended pregnancy during antenatal care visit and incorporate the promotion of mental health into their national reproductive and child health programme.

## Ethical approval and consent

Our study is based on a secondary dataset with no identifiable information on the survey participants. This dataset is available in public domain for research use and hence no approval was required from any institutional review board. The data can be downloaded from the website of the United Kingdom Data Archives University of Essex after creating account (https://www.ukdataservice.ac.uk/). The data for the current study was downloaded from the afore-mentioned website after taking permission (I.D. No. 90978).

## Consent to publish

Not applicable.

## Availability of data and materials

The data which support our findings is contained within the manuscript.

## Abbreviations

YLS: Young Live Study
WHO: World Health Organization
INFHS: Indian National Family Health Survey
AOR: Adjusted Odds Ratio
CI: Confidence Interval
